# Peloids as Thermotherapeutic Agents

**DOI:** 10.3390/ijerph18041965

**Published:** 2021-02-18

**Authors:** Francisco Maraver, Francisco Armijo, Miguel Angel Fernandez-Toran, Onica Armijo, Jose Manuel Ejeda, Iciar Vazquez, Iluminada Corvillo, Silvia Torres-Piles

**Affiliations:** 1 Medical Hydrology Group, Department of Radiology, Rehabilitation & Physiotherapy, Complutense University of Madrid, Plaza Ramón y Cajal s/n, 28040 Madrid, Spain; farmijoc@ucm.es (F.A.); jmejeda@ucm.es (J.M.E.); i.vazquez@igme.es (I.V.); 2 Professional School of Medical Hydrology, Complutense University of Madrid, Plaza Ramón y Cajal s/n, 28040 Madrid, Spain; corvillo@ucm.es; 3 Balneario de Cofrentes, Calle Balneario s/n, 46625 Valencia, Spain; mangel@balneario.com; 4 La Paz University Hospital, Universidad Autonoma de Madrid, Paseo de la Castellana 261, 28046 Madrid, Spain; onica.armijo@gmail.com; 5 Geological Survey of Spain (IGME), Calle de la Calera 1, 28760 Madrid, Spain; 6 Research Group in Immunophysiology, Department of Medical-Surgical Therapy, Faculty of Medicine, University of Extremadura, Avda. Elvas s/n, 06071 Badajoz, Spain; storres@unex.es

**Keywords:** peloid, thermotherapy, mud therapy, pelotherapy, clay, peat, microcrystalline cellulose, thermal flow, instrumental texture

## Abstract

The use of peloids as heat-providing therapeutic systems dates back to antiquity. Such systems consist of a liquid phase and an organic or inorganic solid phase. The latter facilitates the handling, preparation and stability of the solid–liquid system, modifying its organoleptic and phy-sicochemical properties, and improves its efficacy and tolerance. Peloids enable the application of heat to very specific zones and the release of heat at a given rate. The aims of this work are to study 16 reference peloids used in medical spa centers as thermo-therapeutic agents as well as to propose nine raw materials as a solid phase for the preparation of peloids. The physical properties studied are the centesimal composition, the instrumental texture and the thermal parameters. In conclusion, the peloids of the medical spas studied are used as thermotherapeutic agents in the treatment of musculoskeletal disorders, especially in knee osteoarthritis and to a lesser extent in back pain and psoriatic arthropathy. The clinical experience in these centers shows that the main effects of the application of their peloids are the reduction of pain, an increase in the joint’s functional capacity and an improvement in the quality of life. As thermotherapeutic agents, all the peloids of the me-dical spas studied and the pastes (raw materials with distilled water) examined showed a heat flow rate of up to four times lower than that shown by the same amount of water. The raw materials studied can be used as solid phases for the preparation of peloids with mineral waters.

## 1. Introduction

Since ancient times, peloids have been used as heat-providing healing systems [1]. Currently, peloid therapy is used in health resort medicine in the form of both balneothe-rapy and thalassotherapy.

Since the middle of the last century, this technique has been referred to as mud or pelotherapy. A peloid is defined as “a mature mud or mud suspension or dispersion with curative or cosmetic properties, consisting of a complex mixture of fine grained materials of geological and/or biological origin, mineral or sea water, and organic compounds commonly arising from some biological metabolic activity” [2].

In physicochemical terms, peloids may be considered heterogeneous systems with a solid phase comprised of a mixture of organic and/or inorganic solids, suspended or humidified, with a liquid phase consisting of a solution of ions and molecules of inorganic and organic origin whose solvent is water [3]. The term “system” is used to describe the specific proportion of material that contains defined amounts of one or more substances, ordered in one or more phases. The term “phase” refers to a homogeneous and physically different component of a system separated from the other components by defined boun-dary surfaces [4].

The main properties of peloids recognised are: their applications in thermotherapy [2,3,5,6] manifested by biological effects, metabolic and enzymatic activity, vascular, neuromuscular, analgesic and modifications of the viscoelastic properties of the tissues [7,8]; several other studies have confirmed their anti-inflammatory [9,10,11,12,13,14], chondroprotective [10,11] and immunological actions [10,14,15,16] which can be attributed to their chemical composition [17,18,19,20] and organic content [21,22,23,24,25,26].

During the use of peloids in thermotherapy, heat acts as a therapeutic agent. While mainly contained in the liquid phase, this heat is moderated by the solid phases such that it can be applied to very specific zones at a predetermined release rate. In thermodynamic terms, heat transmission is a so-called irreversible phenomenon [27]. Irreversible processes exist whereby there is transport of some physical magnitude from one region to another of a system due to a gradients of different physical magnitudes; such processes are known as transport phenomena and can be expressed through phenomenological laws [28].

Transport phenomena originate from a series of causes, such as the temperature gradient. These magnitudes are designated “forces” in the thermodynamics of irreversible processes. These “forces” give rise to “fluxes” such as heat flow [29].

Several phenomenological equations exist to describe transport phenomena as proportions, such as Fourier’s law, between the thermal flow (Φ) and temperature gradient. The heat equation is a mathematical model that describes the temperature changes that a solid body goes through, which can be summarised as the calorific energy flowing from zones of a greater to lower temperature, and that this energy is proportional to the temperature gradient between the two zones. Accordingly, a greater temperature gradient is needed for a peloid to achieve the best thermotherapeutic effects, provided they are well-tolerated by the patient without producing any undesirable side effects.

This determines the important role of the solid peloid phase that acts as a vehicle or coadjuvant, improving the efficacy of the therapeutic agent. The final goal is the sustained release of heat. The diffusion velocity, together with the biocompatibility, are the most important factors to consider when selecting a predetermined solid phase.

Peloids need to have three qualities: they should have a low cooling speed, should be easy to handle, and should offer a pleasant feeling when applied to the skin. They are used in full or partial baths or applied locally to a given skin zone at a temperature of 42 to 45 °C in 1 to 2 cm layers in 20 to 30 min sessions [30].

According to several clinical studies, the most effective indications of this form of therapy are musculoskeletal disorders of the knee [13,31,32,33,34,35,36,37,38,39,40,41,42,43], spine [44,45,46,47,48,49,50], hand [51,52,53,54], as well as fibromyalgia syndrome [55,56,57,58], carpal tunnel syndrome and chronic lateral epicondylitis [59,60].

The mechanical and thermal properties of clays and peats that make them useful for thermotherapy have been well described in the literature [30,61,62,63,64,65,66,67,68,69,70,71,72,73,74,75,76,77,78,79,80,81,82,83,84,85,86,87,88,89]. The aims of this study are, firstly, to examine the physical properties, heat and texture, of 16 reference peloids used in medical spas (MSs); secondly, to determine the physical characteristics of nine raw materials (RMs)—six inorganic (clays) and three organic (two peats and a microcrystalline cellulose); and thirdly, to study the properties of the pastes obtained with these nine RMs mixed with distilled water (RM/DW), comparing them with the MSs.

## 2. Materials and Methods

### 2.1. Materials

#### 2.1.1. Peloids Used in Medical Spas (MSs)

**Figure 1:**
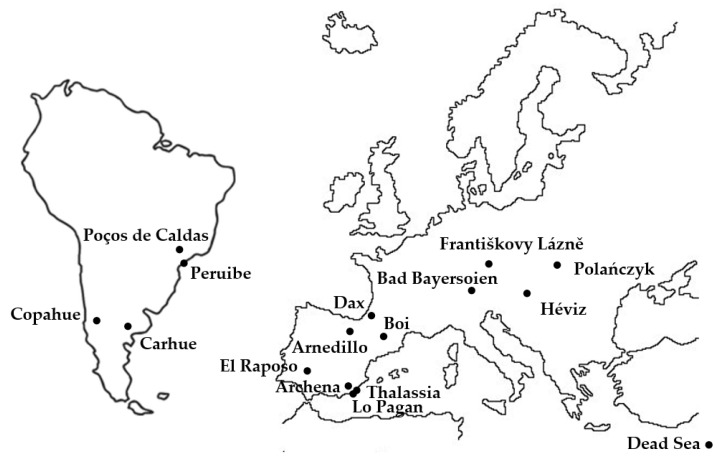
Origins of the peloids used in medical spas (MSs).

The origins of the 16 MS samples were Carhué and Copahue (Argentina), Peruibe and Poço de Caldas (Brazil), Františkovy Lázně (Czechia), Terdax (France), Bad Bayersoien (Germany), Hévíz (Hungary), Dead Sea (Israel), Polanczyk (Poland) and Archena, Arnedillo, Caldes de Bohí, El Raposo, Lo Pagan and Thalassia (Spain)—see Figure 1.

#### 2.1.2. Raw Materials (RMs)

The RMs examined were six inorganic clays lacking carbon chains in their structure and three organic materials—two peats and one microcrystalline cellulose material. These materials and their suppliers were: aluminium bentonite from Süd Chemie Spain (C1), magnesium bentonite from Süd Chemie Spain (C2), kaolin from Avisa (C3), kerolite from Süd Chemie Spain (C4), palygorskite from Tolsa (C5), sepiolite from Tolsa (C6), blonde peat from Plantaflor (P7), milled peat from Turberas del Buyo y del Gistral (P8) and microcrystalline cellulose Avicel PH 101 from FMC Europa (MC9), proposed for the first time as a solid phase.

### 2.2. Methods

#### 2.2.1. Centesimal Composition

The centesimal composition was quantified by desiccation at 105 °C in an oven until constant weight, and expressed as a percentage relative to the whole peloid. The water content was calculated by the difference with respect to the percentage of solids. Ash is the residue of the solid components left behind after incineration at 850 °C in a muffle furnace until constant weight, expressed as a percentage (weight-to-weight) relative to the whole peloid. The lower the ratio of the percentage of ash to the percentage of solids, the lower the materials’ content of substances that are volatile or removable by high tempe-ratures [3,90]. Knowing the centesimal composition makes it possible to deduce the type of solid phase that constitutes the peloid (inorganic or organic) and calculate its specific heat.

#### 2.2.2. Instrumental Texture

The texture was determined using a Brookfield Texture Analyzer model LRFA 1000 with a round 10 mm stainless steel probe following the Texture Profile Analysis (TPA) method involving two consecutive cycles for each determination. The instrumental texture provides information on hardness, or the force necessary for a given deformation measured in grams (g); adhesiveness, or the work needed to overcome the forces of attraction between the surface of a material and the surfaces in contact with it, measured in grams per second (g.s); and cohesion, defined as the inner bond forces that maintain the shape of the product. This last parameter is the property of non-consolidated fine-grained materials whereby particles remain joined together due to the surface forces. It is adimensional with high values indicating higher cohesion in the product [91,92,93]. Hardness conditions the abrasiveness of the peloid responsible for the higher or lower tolerance on the part of the patient, while adhesiveness and cohesion are decisive when choosing the technique of application of the peloid (ilutation, brushing, bathing).

#### 2.2.3. Thermal Parameters

- Specific heat (C_p_)
  1. Calculation of the (C_p_) of the MSs
    The equation proposed by Armijo et al. [80] with which the specific heat of a peloid can be calculated as a function of its ash (A) and water (W) contents is:
    C_p_ = 1.26023 + 0.02926 (W) − 0.00628 (A) + 0.000063 (W) (A)
  2. Determination of the (C_p_) of the RMs
    The (C_p_)’s of the RMs were determined using an air-cooled differential scanning ca-lorimeter DSC1 (Mettler, Toledo). The characteristics of this system include a temperature accuracy of ±0.02 K and a heating rate of 10 K/min. The (C_p_) capacities of the nine RMs were determined to be between 45 and 36 °C, temperatures that are commonly used for peloid application in spas; the same methods were used to obtain the cooling curves. This takes into account that although the (C_p_) varies across the temperature range, these variations are so small that the mean values may easily be considered with no appreciable error.
  3. Calculation of the (C_p_) of the pastes of the raw material–distilled water mixtures (RM/DW)
    For pastes, the (C_p_) of the system is given by the sum of their components. According to the general equation:
    Cp(P)=[(Si)Cp(Si) + (100− (Si)) Cp(W)]/100
    where (S_i_) is the weight percentage of the different solid constituents and c_p_(S_i_) and c_p_(W) are the specific heat capacities of both solid and water.
    Pastes (RM/DW) were prepared by adding distilled purified water to the RMs and leaving them for 24 h for the water to penetrate, followed by manual mixing. The distilled purified water used to prepare the pastes was obtained using a system consisting of a Fistreen Cyclon distiller fitted to a Water Pro purification system from Labconco and a Sy-nergy UV system from Millipore.
    The (C_p_) is used to calculate the amount of heat (Q) that a peloid or paste can give off over a range of temperatures.
- Cooling curve test
  Cooling curves were prepared by plotting temperature against time. The thermometer used has a Pt 100 probe to measure the product temperature at 15 s intervals from 45 to 36 °C. The equation best fitting the experimental curve was determined using the program Origin 8 [94].
  These curves were then used to obtain the relaxation time (t_r_) defined as the time needed for the temperature to drop exponentially by 37% of its starting value (1/e = 0.37). Accordingly, for a peloid applied at 45 °C and attaining a final temperature of 36 °C, in the first t_r_, the temperature reached would be (36 + 9/e) 39.3 °C. Furthermore, this same time would be required for the temperature to drop to (36 + 9/e2) 37.2 °C, and in the same time intervals to 36.4 °C and 36.1 °C. Over three times its t_r_, the temperature reached would be 36.4 °C. This is the normal user body temperature, so the peloid would no longer have a thermotherapeutic effect [80].
  From (Q) and (tr), the heat flow (**Φ**) is obtained; that is, the speed of the passage of heat from the peloid to the patient.

## 3. Results and Discussion

### 3.1. Peloids Used in Medical Spas (MSs)

The MSs studied correspond to world-renowned health resort medicine located in nine countries where they are applied as thermotherapeutic agents to more than one hundred thousand patients a year. Table 1 contains the main clinical trials of these MSs pu-blished in recent years (Table 1).

It may be observed that they are used mostly in the treatment of disorders of the musculoskeletal system, especially in knee osteoarthritis, and, to a lesser extent, for psoriatic arthritis, back pain, rheumatoid arthritis and for the muscle pain of post-stroke patients. These indications are consistent with those of other peloids used in other important medical spas [35,37,38,40,41,47,48,49,50].

A study on osteoarthritis with laboratory animals was carried out with Peruibe’s peloid [109].

On the other hand, and although not as thermo-therapeutic, given the special climatic conditions of the thermal stations of Copahue and the Dead Sea, its peloids are also used in dermatological disorders. [110,111,112,113,114,115].

Given the importance of these MSs, both the solid and liquid phases of some have been studied, such as Copahue [68,115,116,117,118], Peruibe [119], Dax [72] and Archena, Arnedillo, Caldes de Bohí, El Raposo and Lo Pagan [17,74,120,121,122,123]; and organic matter, such as Héviz [124,125,126] and the Dead Sea [21,127,128].

The centesimal water, solids and ash contents of the 16 MSs are provided along with their ash:solids ratios in Table 2. These ratios indicate that the first four peloids (Františkovy Lázně, Polańczyk, Caldes de Boí and Bad Bayersoien) have peat as their solid phases while the last 10 show the presence of inorganic materials. The peloid from Héviz originates from the peat base of a lake at a depth of 38 m, which could explain its intermediate ash/solids value [124]. The Copahue peloid shows a reduced ash content due to the presence of sulphur compounds given its volcanic origin. This was confirmed by the odor emitted during the incineration of this peloid [115].

Table 3 lists the values of instrumental texture, hardness, adhesiveness and cohesion of the different spa peloids ordered according to their adhesiveness/hardness ratio.

Those of greater adhesiveness from El Raposo, Lo Pagan, Arnedillo and the Dead Sea could be used for brush applications. Owing to their greater cohesion, the peloids of Poço de Caldas, Thalassia and Carhué would be especially useful for specific body zones. Ge-nerally speaking, those of lower hardness are less abrasive for the patient as there is good correlation between these two parameters [74,121].

As we stated in the introduction and methods, the main therapeutic action of the peloids in rheumatic disorders is due to the action of heat and the way in which they are applied; the most important factor is the amount of water that the peloid contains due to its high values of (C_p_) heat flow (Φ), the amount of heat (Q) that passes in the unit of time toward the temperature drop.

In Table 4, we provide (C_p_) data obtained from the analysis of the percentage of water and ash composition of the MSs, along with the t_r_ and Q released by 1 kg of peloid as the temperature drops from 45 to 39.3 °C (t_r_ temperature), and Φ from the start of the application until the t_r_ is reached.

The higher t_r_ were recorded for the peloids based on organic material, peat, and those used in the Františkovy Lázně, Polanczyk, Bad Bayersoien and Boi spas, meaning they release their heat more slowly than those prepared with inorganic solid phases. The (C_p_) values for the peloids based on peat were in line with those reported in the literature [85].

The Φ values of MSs ranged from 25.1 J/s for the Poço de Caldas peloid to 33.4 J/s for the Arnedillo and Thalassia peloids.

### 3.2. Raw Materials (RMs) and the Pastes (RM/DW)

As we have indicated previously, another of the objectives of this work is to study and proposed RMs that make the elaboration of pastes (RM/DW) possible with mechanical and thermal properties similar to MSs for use as a solid phase for the preparation of peloids as thermotherapeutic agents. We followed the same criteria as for the MSs to achieve this; that is to say, we studied the centesimal composition, instrumental texture and thermal parameters.

Table 2 also shows the centesimal water, solids and ash contents of the RMs. When calculating the ash-to-solids ratio of the solid phase, values close to unity indicate an inorganic product while those approaching zero reflect an organic product, just as for the MSs.

**Figure 2:**
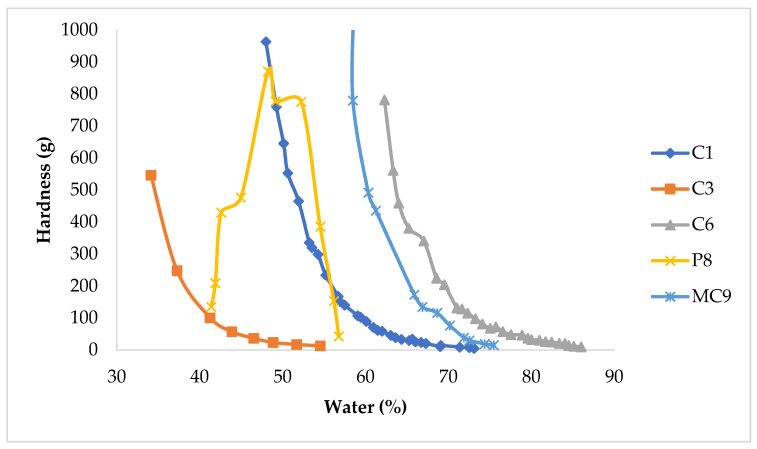
Plots of hardness of the pastes against % water content.

Regarding the instrumental texture of the pastes (RM/DW), the hardness diminishes as their water content rises, following a negative exponential curve. This was not the case for the pastes (RM/DW) based on peat that reached a maximum hardness value. It should be noted that the product MC9, as it is organic, behaved in this regard like a clay, as can be seen in Figure 2. In this figure, we have limited ourselves to including only three inorganic RMs—C1, C3 and C6—and two organic ones—P8 and MC9—for clarity [91].

Table 3 also shows the values of instrumental texture, hardness, adhesiveness, cohesion and the adhesiveness/hardness ratio of the pastes (RM/DW). The pastes (RM/DW) prepared with materials C1 to C6 and MC9 with a hardness of 300 g (we adopted this value because it is the average value of the Spanish peloids we have already studied) previously presented an adhesiveness that ranged from 2481 g in the formulation with C3 to 3672 g for the C1-based formulation, and a hardness of only 1437 g was observed for the organic material MC9. This indicates that the pastes would, overall, show good adhesive properties for the user and pastes (RM/DW) prepared with C3 and MC9 will be more easily removed after their use. The pastes (RM/DW) based on inorganic materials showed much higher ratios than those containing organic materials in the solid phase, as occurs in the MSs. Cohesiveness values are provided for the pastes. The pastes (RM/DW) prepared with inorganic materials showed much higher cohesiveness values than those re-corded for the pastes (RM/DW) containing organic materials in their solid phase, as occurs in the MSs [74].

When water is added to a clay, it arranges itself around the clay particles. If little water is added, clays become coated with water layers, giving rise to a mass with some cohesion. This weak cohesive force between aggregates determines that the clay particles do not slide over each other. This property is made use of when the peloid is applied to the skin for therapeutic purposes [129]. If the water concentrations present in peloids and pastes are reduced, this leads to a lower mobility of bonds and to the products breaking up. As the proportion of water increases, the greater distance between particles causes cohesion loss and the product behaves more or less like a viscous liquid [130].

The adhesiveness/hardness ratios of the pastes (RM/DW) prepared from the inorganic and organic materials and distilled water are shown in Table 3. The inorganic pastes had higher ratios than the pastes made from organic materials, as occurs in the MSs.

Regarding the thermal parameters, the (C_p_) measured from the RM given in J/gK were C1 = 0.859, C2 = 0.981, C3 = 0.972, C4 = 0.870, C5 = 0.765, C6 = 1.253, P7 = 1.291, P8 = 1.148 and MC9 = 1.420 [80].

We also provide the mean (C_p_) data obtained from the pastes (RM/DW) prepared with the inorganic and organic materials in Table 4. These results are in line with those obtained in prior work and, overall, the (C_p_) values of the organic materials were higher than those of the inorganic materials [61,66,71,73,77,81,82,86,131,132].

**Figure 3:**
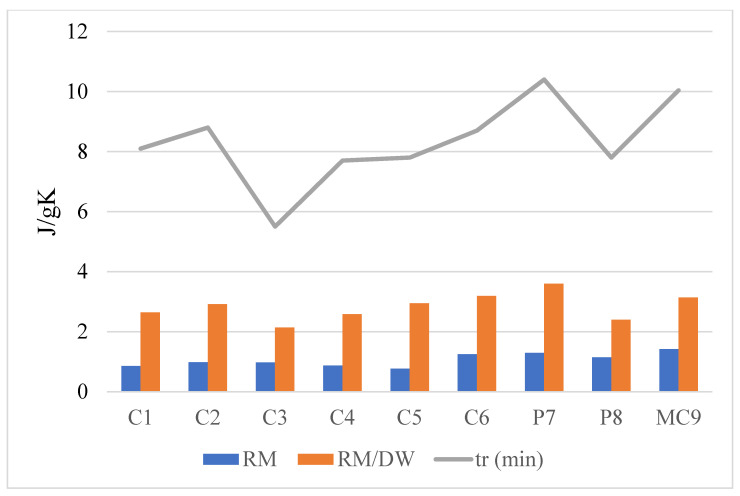
Specific heats of the raw materials (RMs), and specific heats and relaxation times of the raw material–distilled water (RM/DW) formulations.

The highest (C_p_) (expressed in J/gK) was recorded for a paste prepared with the palygorskite clay (C6) and the lowest for a paste prepared with kaolin (C3). The elevated (C_p_) of the preparation based on P7 can be attributed to the large amount of water needed for its preparation (Figure 3).

Table 4 also provides the (C_p_) in J/gK, t_r_ in seconds, the Q in J lost by 1 kg of the MSs and pastes (RM/DW) as it cools from 45 to 39.3 °C, the t_r_ temperature, and the Φ as the J/s reached that temperature range for the MSs and pastes (RM/DW) examined here.

Other works carried out with pastes made with different solid (organic and inorganic) and liquid (DW, natural mineral water and sea water) phases determine the heat transfer curves with temperature ranges that are different from those used [61,66,70,75,83,89,132].

For distilled water, the t_r_ determined under the same conditions as for the MSs and pastes (RM/DW) was 230 s, much lower than the values observed for the MSs and pastes (RM/DW).

According to the t_r_ values in seconds calculated from the heat loss curves for the different pastes, the preparation containing C3 was the fastest at releasing heat, while the slowest in doing so were the C2-based pastes. The pastes containing the organic products P7 and MC9 also released heat slowly.

Corresponding values for the pastes RM/DW were between 29.6 J/s for P8 and 36.9 J/s for the C3-based ones, similar to the values recorded for the MSs.

**Figure 4:**
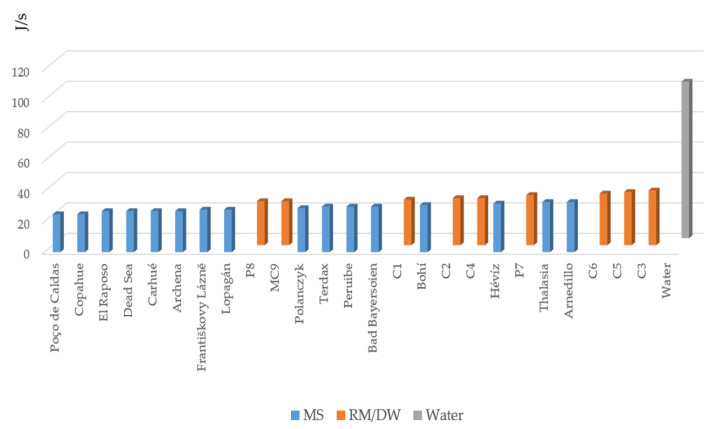
Heat flows recorded for the RM/DW formulations, distilled water and MS.

If we compare the heat-releasing capacity of the MSs and pastes (RM/DW) to that of water, 1 kg of water with a mean (C_p_) of 4.1788 J/kg K cooling in the same conditions as the MSs and pastes (RM/DW) has a t_r_ of 230 s and would release a Q of 23,819 J at a Φ of 103.56 J/s. This means that the solid phases of MSs and pastes (RM/DW) cause the heat to be released three to four times slower than if we applied water directly, which indicates that they can be used on the skin for a thermotherapeutic effect without undesirable effects. Thus, compared to water, the MSs and pastes (RM/DW) enable the application of a large amount of heat over a longer time period in a more localized manner (Figure 4).

## 4. Conclusions

The peloids of the MSs studied, according to the literature cited, are used as thermotherapeutic agents in the treatment of musculoskeletal disorders, especially in knee osteoarthritis and, to a lesser extent, in back pain and psoriatic arthropathy.

The main effects of the application of the MSs studied are the reduction of pain, the increase in the functional capacity of the joint and an improvement in the quality of life.

Considering the centesimal composition of these products, it can be concluded that the ash:solids ratio of the peloids used in the medical spas (MSs) studied, just like the RMs proposed, indicates that a value close to zero corresponds to the organic solid phases, while values close to one correspond to the inorganic phases.

The behavior of the instrumental texture of the pastes (RM/DW) prepared with clays and microcrystalline cellulose is similar to that of the peloids used in medical spas (MSs) with an inorganic solid phase, highlighting that microcrystalline cellulose is an organic product.

As thermotherapeutic agents, all the medical spas (MSs) studied and the pastes exa-mined, both organic and inorganic, showed a heat flow rate of up to four times lower than that shown by the same amount of water. The raw materials studied can be used as solid phases for the preparation of peloids with mineral waters.

## Figures and Tables

**Table 1:** Authors, diagnosis, study design, modalities, application and conclusions of peloids used in medical spas (MSs).

Country Authors (Year), Location of MS	Diagnosis	Study Design	Modalities	Peloid Application, Duration, Number	Conclusions
*Argentina*					
Monasterio and Grenovero (2008), Copahue [95]	Osteoarthritis, Knee and Hand	Prospective	Mud packs and/or General mud bath (Chancho), Thermal baths, Steam	1 or 2 weeks 6 or 12 sessions 20 or 40 min/s	↓ Pain
*Brazil*					
Gouvêa et al. (2021), Peruibe [96]	Osteoarthritis, Knee	Prospective	Muds	9 weeks 45 sessions 20 min/s	↓ Pain ↑ Joint flexibility ↑ Functional capacity
Untura (2008), Poços de Caldas [97]	Osteoarthritis, Knee	Prospective	Muds, Kinesiotherapy, Physical therapy	4 weeks 20 sessions 30–35 min/s	↓ Pain ↑ Joint flexibility ↑ Functional capacity
*Czechia*					
Navrátil et al. (2014), Františkovy Lázně [98]	Temporomandibularjoint disorders	Prospective	Muds, Pulsed magnetic therapy, Laser therapy, Education	10–20 weeks 10 sessions 30 min/s	↓ Pain ↑ Opening ability mouth
*France*					
Forestier et al. (2010), Dax * [32]	Osteoarthritis, Knee	RCT	Muds, Massages, Showers, Pool sessions, Exercise therapy	3 weeks 18 sessions 15 min/s	↓ Pain ↑ Functional capacity
Nguyen et al. (2017), Dax ** [46]	Low back pain	RCT	Muds, Jet showers, Massage, Hot showers, Hydro kinesiotherapy, Education	5 days 6 sessions 15 min/s	NSD Pain NSD Disability NSD Quality of life
*Hungary*					
Gyarmati et al. (2017), Hévíz [52]	Osteoarthritis, Hand	RCT	Muds	3 weeks 15 sessions 20 min/s	↓ Pain ↓ Swollen joints ↑ Hand-grip strength
*Israel*					
Sukenik et al. (1990), Dead Sea [99]	Rheumatoid arthritis	RCT	Mud packs, Sulphur baths	2 weeks 12 sessions 20 min/	↓ Morning stiffness ↑ Hand-grip strength ↑ ADL
Sukenik et al. (1992), Dead Sea [100]	Rheumatoid arthritis	RCT	Mud packs	2 weeks 12 sessions 20 min/	↓ Morning stiffness ↑ Hand-grip strength ↑ ADL
Sukenik et al. (1994), Dead Sea [101]	Psoriatic arthritis	RCT	Mud packs, Sulphur baths, Dead sea water baths, Climatotherapy	3 weeks 18 sessions 20 min/	↓ Morning stiffness ↑ Hand-grip strength ↑ ADL, ↓ PASI
Elkayam et al. (2000), Dead Sea [102]	Psoriatic arthritis	RCT	Mud packs, Sulphur baths, Dead sea water baths, Climatotherapy	4 weeks 24 sessions 20 min/	↓ Morning stiffness ↑ Hand-grip strength ↓ Pain, ↓ PASI
Sukenik et al. (2001), Dead Sea [103]	Psoriatic arthritis and Fibromyalgia	RCT	Mud packs, Sulphur baths, Dead sea water baths, Climatotherapy	4 weeks 24 sessions 20 min/	↓ Active joints ↓ Number tender points ↑ Pain threshold
Flusser et al. (2002), Dead Sea [31]	Osteoarthritis, Knee	Prospective	Mud packs	3 weeks 15 sessions 20 min/	↓ Pain ↑ Functional capacity
Codish et al. (2005), Dead Sea [104]	Rheumatoid arthritis, Hand	RCT	Mud packs	3 weeks 15 sessions 20 min/	↓ Pain ↓ Swollen joints
Abu-Shakra et al. (2014), Dead Sea [105]	Low back pain	RCT	Mud packs	3 weeks 15 sessions 20 min/	↓ Pain ↓ Disability ↓ Flexibility
*Spain*					
Canelas et al. (2010), Archena [106]	Osteoarthritis, Knee	Prospective	Muds, Sulphur baths, Showers, Massages	12 days 12 sessions 30 min/s	↓ Pain ↑ Functional capacity ↓ Drugs
Espejo et al. (2013b), El Raposo [33]	Osteoarthritis, Knee	RCT	Muds, Baths, Thermal jets	11 days 11 sessions 30 min/s	↓ Pain ↑ Quality of life ↓ Drugs
Ortega et al. (2017), El Raposo [11]	Osteoarthritis, Knee	Prospective	Muds, Baths, Thermal jets	10 days 10 sessions 45 min/s	↓ Pain ↑ Functional capacity ↑ Quality of life
Gálvez et al. (2019), El Raposo [13]	Osteoarthritis, Knee	Prospective	Muds, Baths, Thermal jets	10 days 10 sessions 45 min/s	↓ Pain ↑ Functional capacity ↑ Quality of life
Morer et al. (2017), Thalassia [107]	Post-stroke	Prospective	Muds, Aquatic therapy (Halliwick), Climatotherapy	3 weeks 15 sessions 30 min/s	↓ Pain ↑ Balance ↑ Mobility
Morer et al. (2020), Thalassia [108]	Post-stroke	Prospective	Muds, Aquatic therapy (Halliwick), Climatotherapy	2 weeks 10 sessions 30 min/s	↓ Pain ↑ Balance ↑ Mobility

RCT: randomized controlled trial; NSD: not significant difference; ADL: Activities daily living; PASI: Psoriasis area severity index; (*) Dax, Balaruc-les Bains, Aix les Bains; (**) Dax, Saint Amand les-Eaux, Royat, Balaruc-les Bains, Aix les Bains.

**Table 2:** Centesimal composition of peloids used in medical spas (MSs) and raw materials (RMs).

MS and RM	Water (%)	Solids (%)	Ash (%)	Ash/Solids
Františkovy Lázně (Czechia)	83.0	17.0	0.6	0.03
Polańczyk (Poland)	87.1	12.9	0.6	0.04
Caldes Boi (Spain)	84.4	15.6	1.6	0.10
Bad Bayersoien (Germany)	85.6	14.5	1.9	0.13
Copahue (Argentina)	56.2	43.8	16.0	0.36
Héviz (Hungary)	77.7	22.3	10.0	0.45
Dead Sea (Israel)	30.1	70.0	54.2	0.77
Lo Pagan (Spain)	34.3 *	65.7 *	56.2 *	0.85 *
El Raposo (Spain)	39.6 *	60.4 *	53.2 *	0.88 *
Thalassia (Spain)	59.9	40.1	35.8	0.88
Carhue (Argentina)	44.5	55.5	40.0	0.90
Peruibe (Brazil)	58.8	41.2	37.6	0.91
Archena (Spain)	74.6 *	25.4 *	23.3 *	0.92 *
Terdax (France)	46.1 **	53.9 **	50.2 **	0.93 **
Arnedillo (Spain)	31.4 *	68.6 *	64.5 *	0.94 *
Poços de Caldas (Brazil)	52.6	49.8	47.4	0.95
C1	12.55	87.45	81.46	0.93
C2	11.54	88.46	82.70	0.93
C3	1.00	99.00	88.15	0.89
C4	8.3	91.70	85.93	0.94
C5	9.45	90.55	81.32	0.90
C6	10.14	89.86	82.19	0.91
P7	23.89	76.11	2.31	0.03
P8	31.96	68.04	39.37	0.57
MC9	4.07	95.93	0	0

* Data from Maraver et al., 2004 [120]. ** Armijo, 2007 [121]. C1 to MC9 are raw materials (RMs).

**Table 3:** Instrumental texture of the peloids used in medical spas (MSs) and the raw material–distilled water mixtures (RM/DW).

MS and RM/DW	Hardness (g)	Adhesiveness (g s)	Cohesiveness	Adhesiveness/ Hardness
Peruibe (Brazil)	38	1	0.48	0.01
Polańczyk (Poland)	924	763	0.30	0.83
Bad Bayersoien (Germany)	1163	1410	0.23	1.21
Copahue (Argentina)	200	890	0.45	4.45
Františkovy Lázně (Czechia)	105	709	0.56	6.78
Caldes Boi (Spain)	106	909	0.61	8.56
Dead Sea (Israel)	350	3097	0.86	8.84
Héviz (Hungary)	139	1272	0.70	9.14
Arnedillo (Spain)	462 *	4962 *	0.50 *	10.74
Carhue (Argentina)	65	696	0.96	10.76
Poços de Caldas (Brazil)	122	1426	0.99	11.69
Thalassia (Spain)	45	548	0.96	12.10
Lo Pagan (Spain)	461 *	6966 *	0.50 *	15.11
El Raposo (Spain)	394 *	7102 *	0.80 *	18.03
Archena (Spain)	132 *	2491 *	0.80 *	18.87
Terdax (France)	138 *	2646 *	0.65 *	19.17
C1	300	3672	0.96	12.24
C2	300	3216	0.92	10.72
C3	300	2481	0.71	8.27
C4	300	3317	0.88	11.06
C5	300	2950	0.81	9.83
C6	300	3558	0.93	11.86
P7	300	15	0.38	0.05
P8	300	1	0.26	0.003
MC9	300	1437	0.42	4.79

* Data from Armijo, 2007 [121].

**Table 4:** Specific heat (C_p_), relaxation time, heat amount and heat flow recorded for peloids used in medical spas (MSs) and the pastes (RM/DW) with a hardness of 300 g.

MS and RM/DW	C_p_ (J/gK)	t_r_ (s)	Q (J)	Φ (J/s)
Dead Sea (Israel)	1.9	400	10,830	27.1
Arnedillo (Spain)	1.9	324	10,830	33.4
Lo Pagan (Spain)	2.0	400	11,571	28.9
El Raposo (Spain)	2.2	468	12,654	27.0
Terdax (France)	2.4	456	13,908	30.5
Poços de Caldas (Brazil)	2.5	564	14,136	25.1
Carhue (Argentina)	2.8	578	15,789	27.3
Copahue (Argentina)	2.9	648	16,302	25.2
Peruibe (Brazil)	2.9	534	16,416	30.7
Thalassia (Spain)	2.9	498	16,644	33.4
Archena (Spain)	3.4	708	19,437	27.5
Héviz (Hungary)	3.5	624	20,064	32.2
Františkovy Lázně (Czechia)	3.7	744	21,033	28.3
Caldes Boi (Spain)	3.7	684	21,204	31.0
Bad Bayersoien (Germany)	3.8	696	21,432	30.8
Polańczyk (Poland)	3.8	726	21,717	29.9
C1	2.6	486	15,039	30.9
C2	2.9	528	16,638	31.5
C3	2.1	330	12,176	36.9
C4	2.6	462	14,710	31.8
C5	3.0	468	16,814	35.9
C6	3.2	522	18,199	34.9
P7	3.6	624	20,675	33.1
P8	2.4	468	13,834	29.6
MC9	3.1	600	17,906	29.8

## Data Availability

Not applicable.

## References

[1] Maraver F. (2006). Antecedentes históricos de la peloterapia. An. Hidrol. Med..

[2] Gomes C., Carretero M.I., Pozo M., Maraver F., Cantista P., Armijo F., Legido J.L., Teixeira F., Rautureau M., Delgado R. (2013). Peloids and pelotherapy: Historical evolution, classification and glossary. Appl. Clay Sci..

[3] Armijo F. (1991). Propiedades térmicas de los peloides. Bol. Soc. Esp. Hidrol. Med..

[4] Glasstone S. (1974). Textbook of Physical Chemistry.

[5] Gomes C.D.S.F. (2018). Healing and edible clays: A review of basic concepts, benefits and risks. Environ. Geochem. Health.

[6] Carretero M.I. (2020). Clays in pelotherapy. A review. Part II: Organic compounds, microbiology and medical applications. Appl. Clay Sci..

[7] Pastor Vega J.M., Martínez M., Pastor Vega J.M., Sendra F. (1998). Termoterapia. Manual de Medicina Física.

[8] Maraver F., Fernández-Torán M.A., Corvillo I., Morer C., Vázquez I., Aguilera L., Armijo F. (2015). Pelotherapy, a review. Med. Nat..

[9] Giacomino M.I., De Michele D. (2007). ¿Es el fango un antiinflamatorio?. An. Med. Interna.

[10] Fioravanti A., Cantarini L., Guidelli G.M., Galeazzi M. (2011). Mechanisms of action of spa therapies in rheumatic diseases: What scientific evidence is there?. Rheumatol. Int..

[11] Ortega E., Gálvez I., Hinchado M.D., Guerrero J., Martín-Cordero L., Torres-Piles S. (2017). Anti-inflammatory effect as a mechanism of effectiveness underlying the clinical benefits of pelotherapy in osteoarthritis patients: Regulation of the altered inflammatory and stress feedback response. Int. J. Biometeorol..

[12] Cozzi F., Ciprian L., Carrara M., Galozzi P., Zanatta E., Scanu A., Sfriso P., Punzi L. (2018). Balneotherapy in chronic inflammatory rheumatic diseases—A narrative review. Int. J. Biometeorol..

[13] Gálvez I., Torres-Piles S., Ortega E. (2019). Innate/inflammatory bioregulation and clinical effectiveness of whole-body hyperthermia (balneotherapy) in elderly patients with osteoarthritis. Int. J. Hyperth..

[14] Gálvez I., Torres-Piles S., Ortega E. (2020). Effect of mud-bath therapy on the innate/inflammatory responses in elderly patients with osteoarthritis: A discussion of recent results and a pilot study on the role of the innate function of monocytes. Int. J. Biometeorol..

[15] de la Fuente M., Hernández-Torres A. (2014). Modulación inmunológica y envejecimiento en peloterapia. Peloterapia: Aplicaciones Médicas y Cosméticas de Fangos Termales.

[16] Gálvez I., Torres-Piles S., Ortega-Rincón E. (2018). Balneotherapy, Immune System, and Stress Response: A Hormetic Strategy?. Int. J. Mol. Sci..

[17] Carretero M.I., Pozo M., Martín-Rubí J.A., Pozo E., Maraver F. (2010). Mobility of elements in interaction between artificial sweat and peloids used in Spanish spas. Appl. Clay Sci..

[18] Morer C., Roques C.-F., Françon A., Forestier R., Maraver F. (2017). The role of mineral elements and other chemical compounds used in balneology: Data from double-blind randomized clinical trials. Int. J. Biometeorol..

[19] Calin M.R., Radulescu I., Ion A.C., Capra L., Almasan E.R. (2020). Investigations on chemical composition and natural radioactivity levels from salt water and peloid used in pelotherapy from the Techirghiol Lake, Romania. Environ. Geochem. Health.

[20] Gomes C.F., Gomes J.H., Da Silva E.F. (2020). Bacteriostatic and bactericidal clays: An overview. Environ. Geochem. Health.

[21] Nissenbaum A., Rullkötter J., Yechieli Y. (2002). Are the Curative Properties of ‘Black Mud’ from the Dead Sea Due to the Presence of Bitumen (Asphalt) or Other Types of Organic Matter?. Environ. Geochem. Health.

[22] Hanzel A., Berényi K., Horváth K., Szendi K., Németh B., Varga C. (2019). Evidence for the therapeutic effect of the organic content in Szigetvár thermal water on osteoarthritis: A double-blind, randomized, controlled clinical trial. Int. J. Biometeorol..

[23] Calderan A., Carraro A., Honisch C., Lalli A., Ruzza P., Tateo F. (2020). Euganean therapeutic mud (NE Italy): Chlorophyll a variations over two years and relationships with mineralogy and geochemistry. Appl. Clay Sci..

[24] Martínez-Villegas N., Muñoz M.S., González-Hernández P., Rodríguez C.M., Cossio J.B., Díaz R.H., Castillo J.R.F., Rudnikas A.G., López C.D., Pérez-Gramatges A. (2020). Inorganic and organic characterization of Santa Lucía salt mine peloid for quality evaluations. Environ. Sci. Pollut. Res. Int..

[25] Szabó I., Varga C. (2020). Finding possible pharmacological effects of identified organic compounds in medicinal waters (BTEX and phenolic compounds). Int. J. Biometeorol..

[26] Zampieri R.M., Adessi A., Caldara F., Codato A., Furlan M., Rampazzo C., De Philippis R., La Rocca N., Valle L.D. (2020). Anti-Inflammatory Activity of Exopolysaccharides from Phormidium sp. ETS05, the Most Abundant Cyanobacterium of the Therapeutic Euganean Thermal Muds, Using the Zebrafish Model. Biomolecules.

[27] Bird R., Stewart W., Lightfoot E. (2007). Transport Phenomena.

[28] Wei J. (1966). Irreversible thermodynamics in engineering. Ind. Eng. Chem..

[29] Groot S. (1966). Thermodynamics of Irreversible Processes.

[30] Ferrand T., Yvon J. (1991). Thermal properties of clay pastes for pelotherapy. Appl. Clay Sci..

[31] Flusser D., Abu-Shakra M., Friger M., Codish S., Sukenik S. (2002). Therapy With Mud Compresses for Knee Osteoarthritis: Comparison of natural mud preparations with mineral-depleted mud. J. Clin. Rheumatol..

[32] Forestier R., Desfour H., Tessier J.-M., Francon A., Foote A.M., Genty C., Rolland C., Roques C.-F., Bosson J.-L. (2010). Spa therapy in the treatment of knee osteoarthritis: A large randomised multicentre trial. Ann. Rheum. Dis..

[33] Espejo-Antunez L., Caro-Puértolas B., Ibáñez Burgos B., Porto-Payán J.M., Torres-Piles S. (2013). Effects of mud therapy on perceived pain and quality of life related to health in patients with knee osteoarthritis. Reumatol Clin..

[34] Espejo-Antúnez L., Cardero-Durán M.A., Garrido-Ardila E.M., Torres-Piles S., Caro-Puértolas B. (2013). Clinical effectiveness of mud pack therapy in knee osteoarthritis. Rheumatology.

[35] Liu H., Zeng C., Gao S.-G., Yang T., Luo W., Li Y.-S., Xiong Y.-L., Sun J.-P., Lei G.-H. (2013). The effect of mud therapy on pain relief in patients with knee osteoarthritis: A meta-analysis of randomized controlled trials. J. Int. Med. Res..

[36] Tefner I.K., Gaál R., Koroknai A., Ráthonyi A., Gáti T., Monduk P., Kiss E., Kovács C., Bálint G., Bender T. (2013). The effect of Neydharting mud-pack therapy on knee osteoarthritis: A randomized, controlled, double-blind follow-up pilot study. Rheumatol. Int..

[37] Xiang J., Wu D., Li J. (2016). Clinical Efficacy of Mudpack Therapy in Treating Knee Osteoarthritis: A meta-analysis of randomized controlled studies. Am. J. Phys. Med. Rehabil..

[38] Ciani O., Pascarelli N.A., Giannitti C., Galeazzi M., Meregaglia M., Fattore G., Fioravanti A. (2017). Mud-Bath Therapy in Addition to Usual Care in Bilateral Knee Osteoarthritis: An Economic Evaluation Alongside a Randomized Controlled Trial. Arthritis Rheum..

[39] Gay C., Guiguet-Auclair C., Coste N., Boisseau N., Gerbaud L., Pereira B., Coudeyre E. (2020). Limited effect of a self-management exercise program added to spa therapy for increasing physical activity in patients with knee osteoarthritis: A quasi-randomized controlled trial. Ann. Phys. Rehabil. Med..

[40] Hou C., Liang L., Chu X., Qin W., Li Y., Zhao Y. (2020). The short-term efficacy of mud therapy for knee osteoarthritis: A meta-analysis. Medicine.

[41] Király M., Kővári E., Hodosi K., Bálint P.V., Bender T. (2020). The effects of Tiszasüly and Kolop mud pack therapy on knee osteoarthritis: A double-blind, randomised, non-inferiority controlled study. Int. J. Biometeorol..

[42] Rat A.-C., Loeuille D., Vallata A., Bernard L., Spitz E., Desvignes A., Boulange M., Paysant J., Guillemin F., Chary-Valckenaere I. (2020). Spa therapy with physical rehabilitation is an alternative to usual spa therapy protocol in symptomatic knee osteoarthritis. Sci. Rep..

[43] Varzaityte L., Kubilius R., Rapoliene L., Bartuseviciute R., Balcius A., Ramanauskas K., Nedzelskiene I. (2020). The effect of balneotherapy and peloid therapy on changes in the functional state of patients with knee joint osteoarthritis: A randomized, controlled, single-blind pilot study. Int. J. Biometeorol..

[44] Cozzi F., Podswiadek M., Cardinale G., Oliviero F., Dani L., Sfriso P., Punzi L. (2007). Mud-bath treatment in spondylitis associated with inflammatory bowel disease—A pilot randomised clinical trial. Jt. Bone Spine.

[45] Ciprian L., Nigro A.L., Rizzo M., Gava A., Ramonda R., Punzi L., Cozzi F. (2011). The effects of combined spa therapy and rehabilitation on patients with ankylosing spondylitis being treated with TNF inhibitors. Rheumatol. Int..

[46] Nguyen C., Boutron I., Rein C., Baron G., Sanchez K., Palazzo C., Dupeyron A., Tessier J.M., Coudeyre E., Eschalier B. (2017). Intensive spa and exercise therapy program for returning to work for low back pain patients: A randomized controlled trial. Sci. Rep..

[47] Costantino M., Conti V., Corbi G., Marongiu F., Marongiu M.B., Filippelli A. (2019). Sulphurous mud-bath therapy for treatment of chronic low back pain caused by lumbar spine osteoarthritis. Intern. Emerg. Med..

[48] Yücesoy H., Geçmen I., Adıgüzel T., Karagülle M., Karagülle M.Z. (2019). Efficacy of balneological outpatient treatment (hydrotherapy and peloidotherapy) for the management of chronic low back pain: A retrospective study. Int. J. Biometeorol..

[49] Forestier R., Suehs C., Françon A., Marty M., Genevay S., Sellam J., Chauveton C., Forestier F.B.E., Molinari N. (2020). Usual care including home exercise with versus without spa therapy for chronic low back pain: Protocol for the LOMBATHERM’ study, a multicentric randomised controlled trial. Trials.

[50] Cozzi F., Galozzi P., Ciprian L., Zanatta E., Polito P., Oliviero F., Carrara M., Punzi L. (2020). Mud-bath treatment of seronegative spondyloarthritis: Experience at the Euganean Thermal Area. Int. J. Biometeorol..

[51] Fortunati N.A., Fioravanti A., Seri G., Cinelli S., Tenti S. (2016). May spa therapy be a valid opportunity to treat hand osteoarthritis? A review of clinical trials and mechanisms of action. Int. J. Biometeorol..

[52] Gyarmati N., Kulisch Á., Németh A., Bergmann A., Horváth J., Mándó Z., Matán Á., Szakál E., Péter T.S., Szántó D. (2017). Evaluation of the Effect of Hévíz Mud in Patients with Hand Osteoarthritis: A Randomized, Controlled, Single-Blind Follow-Up Study. Isr. Med. Assoc. J..

[53] Aksoy M.K., Altan L., Eröksüz R., Ökmen B.M. (2017). The efficacy of peloid therapy in management of hand osteoarthritis: A pilot study. Int. J. Biometeorol..

[54] Tenti S., Manica P., Cheleschi S., Fioravanti A. (2020). Sulfurous-arsenical-ferruginous balneotherapy for osteoarthritis of the hand: Results from a retrospective observational study. Int. J. Biometeorol..

[55] Fioravanti A., Perpignano G., Tirri G., Cardinale G., Gianniti C., Lanza C.E., Loi A., Tirri E., Sfriso P., Cozzi F. (2007). Effects of mud-bath treatment on fibromyalgia patients: A randomized clinical trial. Rheumatol. Int..

[56] Bazzichi L., Da Valle Y., Rossi A., Giacomelli C., Sernissi F., Giannaccini G., Betti L., Ciregia F., Giusti L., Scarpellini P. (2013). A multidisciplinary approach to study the effects of balneotherapy and mud-bath therapy treatments on fibromyalgia. Clin. Exp. Rheumatol..

[57] Bağdatlı A.O., Donmez A., Eröksüz R., Bahadır G., Turan M., Erdoğan N. (2015). Does addition of ‘mud-pack and hot pool treatment’ to patient education make a difference in fibromyalgia patients? A randomized controlled single blind study. Int. J. Biometeorol..

[58] Eröksüz R., Forestier F.B.E., Karaaslan F., Forestier R., Işsever H., Erdoğan N., Karagülle M.Z., Dönmez A. (2020). Comparison of intermittent and consecutive balneological outpatient treatment (hydrotherapy and peloidotherapy) in fibromyalgia syndrome: A randomized, single-blind, pilot study. Int. J. Biometeorol..

[59] Ökmen B.M., Aksoy M.K., Güneş A., Eröksüz R., Altan L. (2017). Effectiveness of PELOID therapy in carpal tunnel syndrome: A randomized controlled single blind study. Int. J. Biometeorol..

[60] Ökmen B.M., Aksoy M.K., Eröksüz R., Altan L. (2017). Efficacy of peloid therapy in patients with chronic lateral epicondylitis: A randomized, controlled, single blind study. Int. J. Biometeorol..

[61] Cara S., Carcangiu G., Padalino G., Palomba M., Tamaninia M. (2000). The bentonites in pelotherapy: Chemical, mineralogical and technological properties of materials from Sardinia deposits (Italy). Appl. Clay Sci..

[62] Beer A.-M., Grozeva A., Sagorchev P., Lukanov J. (2003). Comparative Study of the Thermal Properties of Mud and Peat Solutions Applied in Clinical Practice. Biomed. Tech..

[63] Veniale F., Barberis E., Carcangiu G., Morandi N., Setti M., Tamanini M., Tessier D. (2004). Formulation of muds for pelotherapy: Effects of “maturation” by different mineral waters. Int. J. Biometeorol..

[64] Mourelle M.L. (2006). Caracterización Termofísica de Peloides para Aplicaciones Termoterapéuticas en Centros Termales. Ph.D. Thesis.

[65] Mourelle M., Medina C., Meijide-Failde R., Soto J.L. (2007). Comportamiento termofisico de los peloides. Bol. Soc. Esp. Hidrol. Med..

[66] Legido J., Medina C., Lourdesmourelle M., Carretero M., Pozo M. (2007). Comparative study of the cooling rates of bentonite, sepiolite and common clays for their use in pelotherapy. Appl. Clay Sci..

[67] Gámiz E., Martín-García J.M., Fernández-González M.V., Delgado G., Delgado R. (2009). Influence of water type and maturation time on the properties of kaolinite–saponite peloids. Appl. Clay Sci..

[68] Baschini M., Pettinari G., Vallés J., Aguzzi C., Cerezo P., López-Galindo A., Setti M., Viseras C. (2010). Suitability of natural sulphur-rich muds from Copahue (Argentina) for use as semisolid health care products. Appl. Clay Sci..

[69] De Zárate J.M.O., Hita J.L., Khayet M., Legido J.L. (2010). Measurement of the thermal conductivity of clays used in pelotherapy by the multi-current hot-wire technique. Appl. Clay Sci..

[70] Rebelo M., Viseras C., Lopez-Galindo A.L., Rocha F., Da Silva E.F. (2011). Rheological and thermal characterization of peloids made of selected Portuguese geological materials. Appl. Clay Sci..

[71] Casás L., Legido J., Pozo M., Mourelle L., Plantier F., Bessières D. (2011). Specific heat of mixtures of bentonitic clay with sea water or distilled water for their use in thermotherapy. Thermochim. Acta.

[72] Knorst-Fouran A., Casás L., Legido J., Coussine C., Bessières D., Plantier F., Lagière J., Dubourg K. (2012). Influence of dilution on the thermophysical properties of Dax peloid (TERDAX^®^). Thermochim. Acta.

[73] Casás L., Pozo M., Gomez C., Pozo E., Bessières L., Plantier F., Legido J. (2013). Thermal behavior of mixtures of bentonitic clay and saline solutions. Appl. Clay Sci..

[74] Pozo M., Carretero M.I., Maraver F., Pozo E., Gómez I., Armijo F., Rubí J.A.M. (2013). Composition and physico-chemical properties of peloids used in Spanish spas: A comparative study. Appl. Clay Sci..

[75] Fernández-González M.V., Martín-García J.M., Delgado G., Párraga J., Delgado R. (2013). A study of the chemical, mineralogical and physicochemical properties of peloids prepared with two medicinal mineral waters from Lanjarón Spa (Granada, Spain). Appl. Clay Sci..

[76] Caridad V., De Zárate J.M.O., Khayet M., Legido J.L. (2014). Thermal conductivity and density of clay pastes at various water contents for pelotherapy use. Appl. Clay Sci..

[77] Carretero M., Pozo M., Legido J., Fernández-González M., Delgado R., Gómez I., Armijo F., Maraver F. (2014). Assessment of three Spanish clays for their use in pelotherapy. Appl. Clay Sci..

[78] Khiari I., Mefteh S., Sánchez-Espejo R., Aguzzi C., López-Galindo A., Jamoussi F., Iborra C. (2014). Study of traditional Tunisian medina clays used in therapeutic and cosmetic mud-packs. Appl. Clay Sci..

[79] Sánchez-Espejo R., Cerezo P., Aguzzi C., López-Galindo A., Machado J., Viseras C. (2015). Physicochemical and in vitro cation release relevance of therapeutic muds “maturation”. Appl. Clay Sci..

[80] Armijo F., Maraver F., Pozo M., Carretero M.I., Armijo O., Fernández-Gonzales M., Corvillo I. (2016). Thermal behaviour of clays and clay-water mixtures for pelotherapy. Appl. Clay Sci..

[81] Mato M.M., Casás L.M., Legido J.L., Gómez C., Mourelle L., Bessières D., Plantier F. (2017). Specific heat of mixtures of kaolin with sea water or distilled water for their use in thermotherapy. J. Therm. Anal. Calorim..

[82] Glavaš N., Mourelle M.L., Gómez C.P., Legido J.L., Šmuc N.R., Dolenec M., Kovač N. (2017). The mineralogical, geochemical, and thermophysical characterization of healing saline mud for use in pelotherapy. Appl. Clay Sci..

[83] Fernández-González M.V., Martín-García J.M., Delgado G., Párraga J., Carretero M.I., Delgado R. (2017). Physical properties of peloids prepared with medicinal mineral waters from Lanjarón Spa (Granada, Spain). Appl. Clay Sci..

[84] Awad M.E., López-Galindo A., Sánchez-Espejo R., El-Rahmany M.M., Viseras C. (2018). Thermal properties of some Egyptian kaolin pastes for pelotherapeutic applications: Influence of particle geometry on thermal dosage release. Appl. Clay Sci..

[85] García-Villén F., Sánchez-Espejo R., Carazo E., Borrego-Sánchez A., Aguzzi C., Cerezo P., Viseras C. (2018). Characterisation of Andalusian peats for skin health care formulations. Appl. Clay Sci..

[86] Pozo M., Armijo F., Maraver F., Zuluaga P., Ejeda J.M., Corvillo I. (2019). Variations in the Texture Profile Analysis (TPA) Properties of Clay/Mineral-Medicinal Water Mixtures for Pelotherapy: Effect of Anion Type. Minerals.

[87] Carretero M.I. (2020). Clays in pelotherapy. A review. Part I: Mineralogy, chemistry, physical and physicochemical properties. Appl. Clay Sci..

[88] Özay P., Karagülle M., Kardeş S., Karagülle M.Z. (2020). Chemical and mineralogical characteristics of peloids in Turkey. Environ. Monit. Assess..

[89] Fernández-González M.V., Carretero M.I., Martín-García J.M., Molinero-García A., Delgado R. (2021). Peloids prepared with three mineral-medicinal waters from spas in Granada. Their suitability for use in pelotherapy. Appl. Clay Sci..

[90] Prát S., Brozek B., Lych S. (1963). Biology and Phisics of peloids. Medical Hydrology.

[91] Armijo F., Maraver F., Carretero M.I., Pozo M., Ramos M., Fernandez-Torán M.A., Corvillo I. (2015). The water effect on instrumental hardness and adhesiveness of clay mixtures for pelotherapy. Appl. Clay Sci..

[92] Nishinari K., Kohyama K., Kumagai H., Funami T., Bourne M.C. (2013). Parameters of Texture Profile Analysis. Food Sci. Technol. Res..

[93] Peleg M. (2019). The instrumental texture profile analysis revisited. J. Texture Stud..

[94] Rambaud A., Rambaud J., Berger G., Pauvert B. (1986). Mesure et étude du comportement thermique des boues thermales. J. Fr. Hydrol..

[95] Monasterio A.M., Grenovero S., Legido J.L., Mourelle M.L. (2008). Influencia del Tratamiento termal (Fan/Hidro) en pacientes con diagnóstico de osteo-artrosis primaria de rodilla y manos derivados por el plan Termalismo al complejo termal de Copahue, Neuquén, Argentina en la temporada 2006–2007. Investigaciones En El Ámbito Iberoamericano Sobre Peloides Termales.

[96] Gouvêa P.F.M., Britschka Z.M.N., Gomes C.d.O.M.S., Queiroz N.G.T.d., Salvador P.A.V., Silva P.S.C. (2021). Evaluation of the Use of Sterilized and Non-Sterilized Peruibe Black Mud in Patients with Knee Osteoarthritis. Int. J. Environ. Res. Public Health.

[97] Filho M.U., Legido J.L., Mourelle M.L. (2008). Fangoterapia en las articulaciones periféricas. Mecanismo de acción. Modelo de Protocolo Terapéutico en Artrosis de Rodilla. Investigaciones En El Ámbito Iberoamericano Sobre Peloides Termales.

[98] Navrátil L., Navratil V., Hajkova S., Hlinakova P., Dostalova T., Vranová J. (2014). Comprehensive treatment of temporomandibular joint disorders. Cranio.

[99] Sukenik S., Buskila D., Neumann L., Kleiner-Baumgarten A., Zimlichman S., Horowitz J. (1990). Sulphur bath and mud pack treatment for rheumatoid arthritis at the Dead Sea area. Ann. Rheum. Dis..

[100] Sukenik S., Buskila D., Neumann L., Kleiner-Baumgarten A. (1992). Mud pack therapy in rheumatoid arthritis. Clin. Rheumatol..

[101] Sukenik S., Giryes H., Halevy S., Neumann L., Flusser D., Buskila D. (1994). Treatment of psoriatic arthritis at the Dead Sea. J. Rheumatol..

[102] Elkayam O., Ophir J., Brener S., Paran D., Wigler I., Efron D., Even-Paz Z., Politi Y., Yaron M. (2000). Immediate and delayed effects of treatment at the Dead Sea in patients with psoriatic arthritis. Rheumatol. Int..

[103] Sukenik S., Baradin R., Codish S., Neumann L., Flusser D., Abu-Shakra M., Buskila D. (2001). Balneotherapy at the Dead Sea area for patients with psoriatic arthritis and concomitant fibromyalgia. Isr. Med. Assoc. J..

[104] Codish S., Abu-Shakra M., Flusser D., Friger M., Sukenik S. (2005). Mud compress therapy for the hands of patients with rheumatoid arthritis. Rheumatol. Int..

[105] Abu-Shakra M., Mayer A., Friger M., Harari M. (2014). Dead Sea mud packs for chronic low back pain. Isr. Med. Assoc. J..

[106] Canelas O., Olabe P., Ovejero L., Fernandez-Jaen T. (2010). Estudio prospectivo de 104 pacientes con gonartrosis sometidos a la cura terminal de Archena. Seguimiento a 6 meses. Bol. Soc. Esp. Hidrol. Med..

[107] Morer C., Boestad C., Zuluaga P., Alvarez-Badillo A., Maraver F. (2017). Effects of an intensive thalassotherapy and aquatic therapy program in stroke patients. A pilot study. Rev. Neurol..

[108] Morer C., Michan-Doña A., Alvarez-Badillo A., Zuluaga P., Maraver F. (2020). Evaluation of the Feasibility of a Two-Week Course of Aquatic Therapy and Thalassotherapy in a Mild Post-Stroke Population. Int. J. Environ. Res. Public Health.

[109] Britschka Z.M.N., Teodoro W.R., Velosa A.P.P., De Mello S.B.V. (2007). The efficacy of Brazilian black mud treatment in chronic experimental arthritis. Rheumatol. Int..

[110] Halevy S., Sukenik S. (1998). Different Modalities of Spa Therapy for Skin Diseases at the Dead Sea Area. Arch. Dermatol..

[111] Matz H., Orion E., Wolf R. (2003). Balneotherapy in dermatology. Dermatol. Ther..

[112] Katz U., Shoenfeld Y., Zakin V., Sherer Y., Sukenik S. (2012). Scientific Evidence of the Therapeutic Effects of Dead Sea Treatments: A Systematic Review. Semin. Arthritis Rheum..

[113] Ubogui J., Stengel F.M., Kien M.C., Sevinsky L., Lupo L.R. (1998). Thermalism in Argentina. Alternative or complementary dermatologic therapy. Arch. Dermatol..

[114] Ubogui J., Roma A., Garvier V., García F., Perrotta G., Monasterio A. (2007). Seguimiento clínico de pacientes con psoriasis en las Termas de Copahue (Neuquén-Argentina). An. Hidrol. Med..

[115] Monasterio A.M., Armijo F., Maraver F., Tassi F., Vaselli O., Caselli A. (2016). Therapeutic Effects of the Mineral Waters from Copahue Spa. Copahue Volcano. Active Volcanoes of the World.

[116] Monasterio A.M., de Michele D., Untura Filho M., Giacomino M., Balderrain A. (2008). Termas de Copahue. El Termalismo Argentino.

[117] Baschini M., Soria C.O., Pettinari E., Gamboa M., Sanchez M., Roca Jalil M.E., Soria C.O., Roca Jalil M.E., Vela M.L. (2018). Fangos de Copahue: Una visión desde la ciencia. Copahue: La Ciencia, Lo Mágico Y El Arte De Curar.

[118] Roca Jalil M.E., Sanchez M., Pozo M., Soria C.O., Vela L., Gurnik N., Baschini M. (2020). Assessment of natural and enhanced peloids from the Copahue thermal system (Argentina): Effects of the drying procedure on lidocaine adsorption. Appl. Clay Sci..

[119] da Silva P.S.C., Torrecilha J.K., Gouvea P.F.D.M., Máduar M.F., de Oliveira S.M.B., Scapin M.A. (2015). Chemical and radiological characterization of Peruíbe Black Mud. Appl. Clay Sci..

[120] Maraver F., Corvillo I., Palencia V., Armijo F. Therapeutic muds in Spain. Proceedings of the 3rd Symposium on Thermal Muds in Europe.

[121] Armijo O. (2007). Estudio de los Peloides Españoles. Ph.D. Thesis.

[122] Maraver F., Armijo O., Armijo F., Cendrero A., Gómez J., Fernandez P.L., Quindos L.S., Ródenas C., Sainz C. (2008). Los peloides españoles: En la Catedra de Hidrología Médica. Contribuciones Científicas en Memoria del Profesor Dr. Jesús Soto Torres.

[123] Ovejero L., Ovejero P. (2017). Tratamiento integral del paciente reumático en un balneario. Bol. Soc. Esp. Hidrol. Med..

[124] Gerencsér G., Murányi E., Szendi K., Varga C. (2010). Ecotoxicological Hungarian peloids (medicinal muds). Appl. Clay Sci..

[125] Gerencsér G., Szendi K., Berényi K., Varga C. (2014). Can the use of medical muds cause genotoxicity in eukaryotic cells? A trial using comet assay. Environ. Geochem. Health.

[126] Szendi K., Gerencsér G., Murányi E., Varga C. (2012). Mutagenic activity of peloids in the Salmonella Ames test. Appl. Clay Sci..

[127] Artymuk N.V., Kira E.F., Kondratieva T.A. (2010). Intravaginal gel prepared from Dead Sea peloid for treating luteal-phase defect. Int. J. Gynecol. Obstet..

[128] Spilioti E., Vargiami M., Letsiou S., Gardikis K., Sygouni V., Koutsoukos P., Chinou I., Kassi E., Moutsatsou P. (2016). Biological properties of mud extracts derived from various spa resorts. Environ. Geochem. Health.

[129] Fernández-Torán M.A. (2014). Propiedades Físico-Químicas de Materiales Susceptibles de ser Utilizados en la Preparación de Peloides. Ph.D. Thesis.

[130] Martıánez-Cortizas A., Pontevedra-Pombal X., Garcıáa-Rodeja E., Nóvoa-Muñoz J.C., Shotyk W. (1999). Mercury in a Spanish Peat Bog: Archive of Climate Change and Atmospheric Metal Deposition. Science.

[131] Pozo M., Armijo F., Maraver F., Ejeda J.M., Pozo E., Corvillo I. (2018). Texture profile analysis (TPA) of clay/seawater mixtures useful for peloid preparation: Effects of clay concentration, pH and salinity. Appl. Clay Sci..

[132] Hernández A.C., Awad M.E., Meléndez W., González G., López-Galindo A., Sánchez-Espejo R., García-Villén F., Viseras C. (2019). Colloidal and Thermal Behaviors of Some Venezuelan Kaolin Pastes for Therapeutic Applications. Minerals.

